# Sensor-Enabled Palatal Ramp Prosthesis for Neuromuscular Re-education Following Hemimandibulectomy: A Case Report

**DOI:** 10.7759/cureus.115570

**Published:** 2026-08-31

**Authors:** Sainath S Karad, Amrita Pandita Bhatia, Manisha Kulkarni, Shantanu More, Mayuri Pardeshi

**Affiliations:** 1 Department of Prosthodontics and Crown and Bridge, Late Shri Yashwantrao Chavan Memorial Medical and Rural Development Foundation's Dental College and Hospital, Ahilyanagar, IND

**Keywords:** biofeedback, hemimandibulectomy, mandibular deviation, maxillofacial prosthetics, neuromuscular re-education, palatal ramp prosthesis, prosthodontics, sensor-assisted rehabilitation

## Abstract

Hemimandibulectomy may result in mandibular deviation toward the resected side due to loss of mandibular continuity and unopposed muscular forces, leading to impaired mastication, speech difficulties, and difficulty achieving functional occlusion. Conventional prosthodontic rehabilitation using guide flange and palatal ramp prostheses can assist mandibular guidance but relies on patient compliance and repeated functional training. This case report describes the rehabilitation of a 58-year-old male patient who presented with severe mandibular deviation following right-sided hemimandibulectomy performed for squamous cell carcinoma. A maxillary palatal ramp prosthesis incorporating a touch sensor-based wireless auditory feedback system was fabricated to provide immediate auditory feedback during mandibular guidance exercises. The sensor-enabled prosthesis was used as a temporary training appliance for six months to facilitate mandibular guidance and neuromuscular re-education. At baseline, mandibular deviation was approximately 15 mm from the facial midline, and the maximum mouth opening was 25 mm. Following the six-month rehabilitation period, the patient demonstrated improved mandibular guidance and functional performance. At the final 18-month follow-up, mandibular deviation was 5 mm, and the maximum mouth opening was 32 mm. Improvement in intercuspation, mastication, and speech was also clinically observed. The sensor-enabled appliance remained functional throughout the six-month training period, and no device-related complications were observed. The sensor-assisted palatal ramp was associated with improvement in mandibular guidance and functional measures in this patient. The appliance served as a temporary rehabilitation and training device rather than a definitive prosthesis. As this was a single-patient, uncontrolled case report, the findings should be considered preliminary, and further clinical evaluation is required to determine the effectiveness and generalizability of this approach.

## Introduction

Hemimandibulectomy is commonly performed for the management of oral malignancies, aggressive benign lesions, and severe maxillofacial trauma. Loss of mandibular continuity frequently results in deviation of the residual mandibular segment toward the surgical side due to the unopposed action of the remaining musculature. Consequently, patients experience compromised mastication, impaired speech, altered facial appearance, and difficulty achieving functional occlusion [1-7].

Various prosthodontic treatment modalities have been proposed to minimize mandibular deviation and improve functional rehabilitation. Guide flange prostheses and palatal ramp prostheses are among the most commonly employed methods for retraining mandibular movement and assisting the residual mandible toward a reproducible occlusal position [8-12]. A guide flange provides mechanical guidance through contact with the mandibular teeth, whereas a palatal ramp provides a guiding surface that assists the residual mandibular segment in achieving a more favorable occlusal relationship. The choice of prosthetic rehabilitation depends on the extent of mandibular resection, remaining dentition, functional requirements, and patient-related factors.

Although these approaches have demonstrated clinical success, their effectiveness depends largely on patient motivation, compliance, and repeated functional training [3,7]. In patients with marked mandibular deviation, additional feedback during functional exercises may help the patient recognize the desired mandibular position and repeatedly practice the corrected movement. Recent advances in sensor technology and wireless communication systems have created opportunities for incorporating biofeedback mechanisms into conventional prosthodontic therapy. In the present approach, a touch sensor incorporated into the palatal ramp detects contact with the mandibular teeth at the predetermined guiding position and activates an external buzzer, providing immediate auditory feedback to the patient. This feedback is intended to serve as a positional training cue during mandibular guidance exercises rather than as a quantitative measurement of mandibular movement. This case report describes the clinical use of a sensor-assisted palatal ramp prosthesis as a temporary six-month rehabilitation and training appliance following hemimandibulectomy.

## Case presentation

A 58-year-old male patient reported to the Department of Prosthodontics, Crown and Bridge, Shri Yashwantrao Chavan Memorial Medical and Rural Development Foundation's Dental College and Hospital, Ahilyanagar, Maharashtra, India, with the chief complaint of deviation of the jaw toward the right side and difficulty in chewing and speaking for the past year. The patient had undergone a right-sided hemimandibulectomy on June 12, 2022, for the management of squamous cell carcinoma. No neck dissection or postoperative radiotherapy had been performed. The patient had a history of tobacco chewing four to five times daily for approximately 15-20 years before surgery.

Extraoral examination revealed facial asymmetry with severe deviation of the mandible toward the resected side during opening and closing movements. The maximum mouth opening was approximately 25 mm, and the mandibular deviation measured approximately 15 mm from the facial midline. The patient demonstrated difficulty in achieving a stable intercuspal position, resulting in impaired mastication and speech. The pre-treatment extraoral appearance and mandibular deviation are shown in Figure 1.

**Figure 1:**
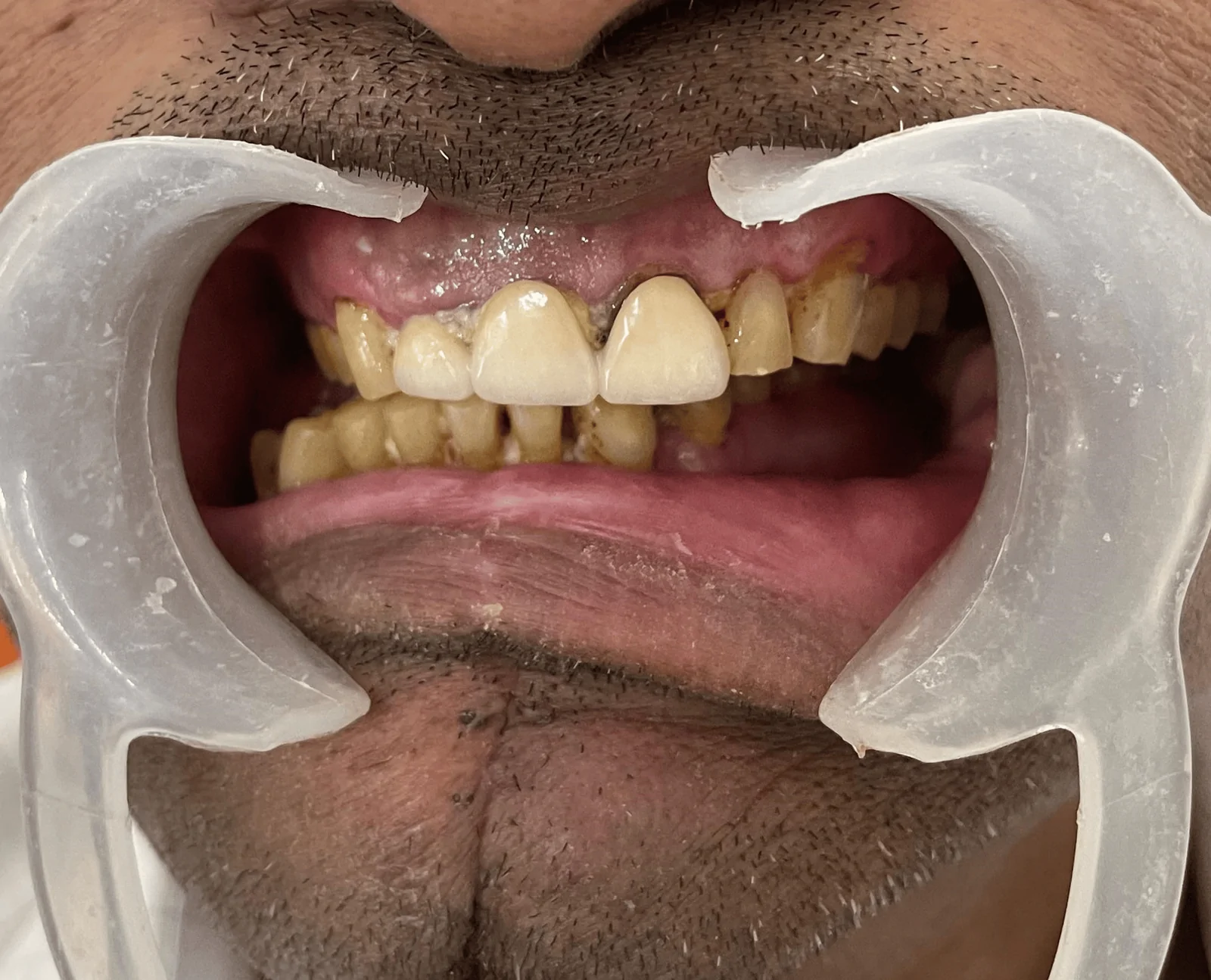
Pre-treatment extraoral view demonstrating mandibular deviation toward the resected side.

Intraoral examination revealed a partially edentulous mandibular arch with missing teeth 45, 46, and 47. The remaining mandibular teeth and surrounding oral structures were evaluated to assess the available occlusal contacts and determine the feasibility of prosthodontic mandibular guidance. The intraoral condition of the partially edentulous mandibular arch is shown in Figure 2.

**Figure 2:**
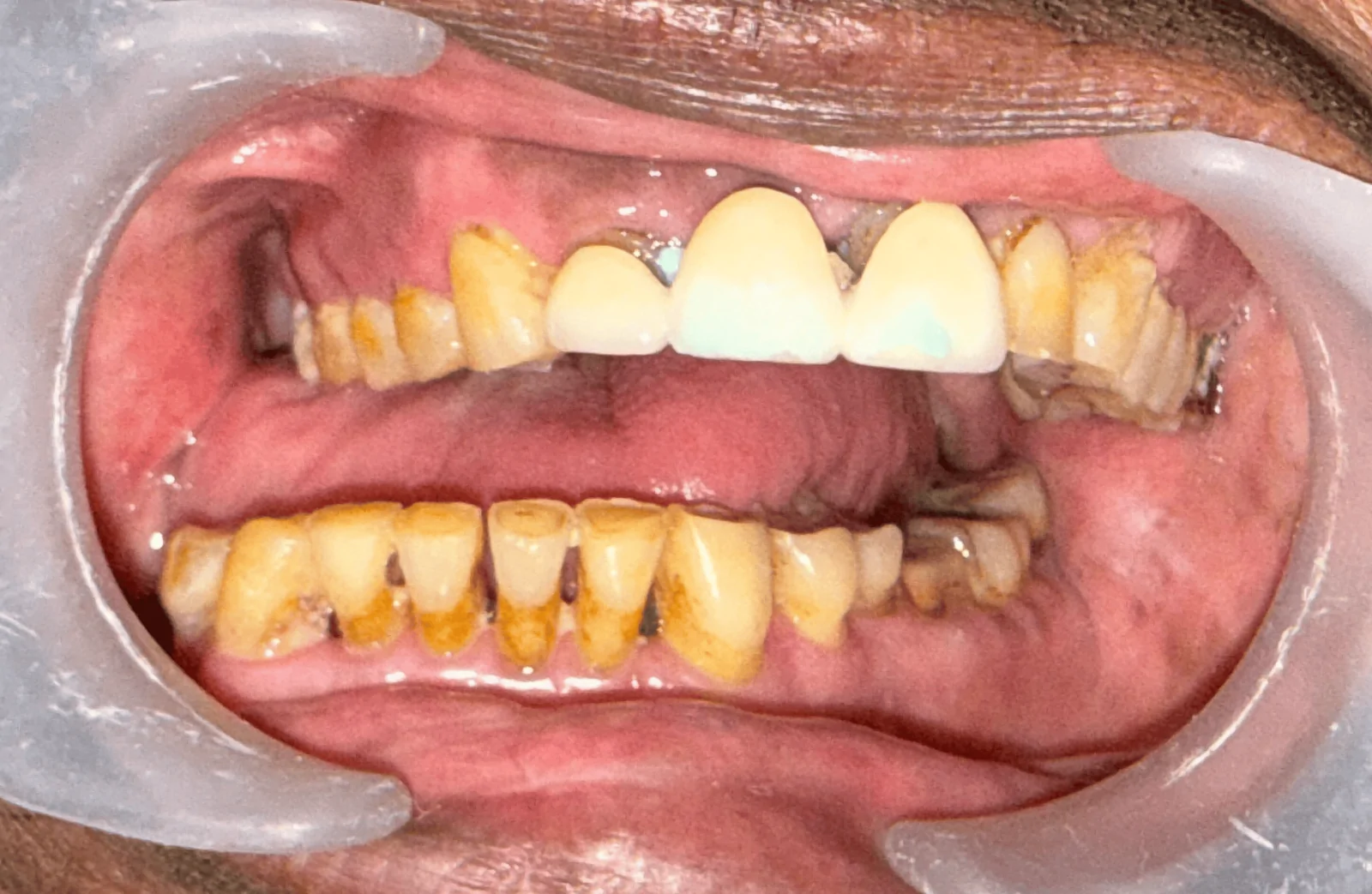
Intraoral view demonstrating the partially edentulous mandibular arch.

The surgical defect was classified as a Cantor and Curtis Class II mandibular defect [10,13]. Based on the clinical findings and functional limitations, rehabilitation with a maxillary palatal ramp prosthesis incorporating a sensor-assisted auditory biofeedback system was planned to facilitate neuromuscular re-education and improve mandibular guidance. Preliminary impressions of both arches were made using irreversible hydrocolloid impression material, and diagnostic casts were obtained. The maxillary and mandibular preliminary impressions are shown in Figure 3.

**Figure 3:**
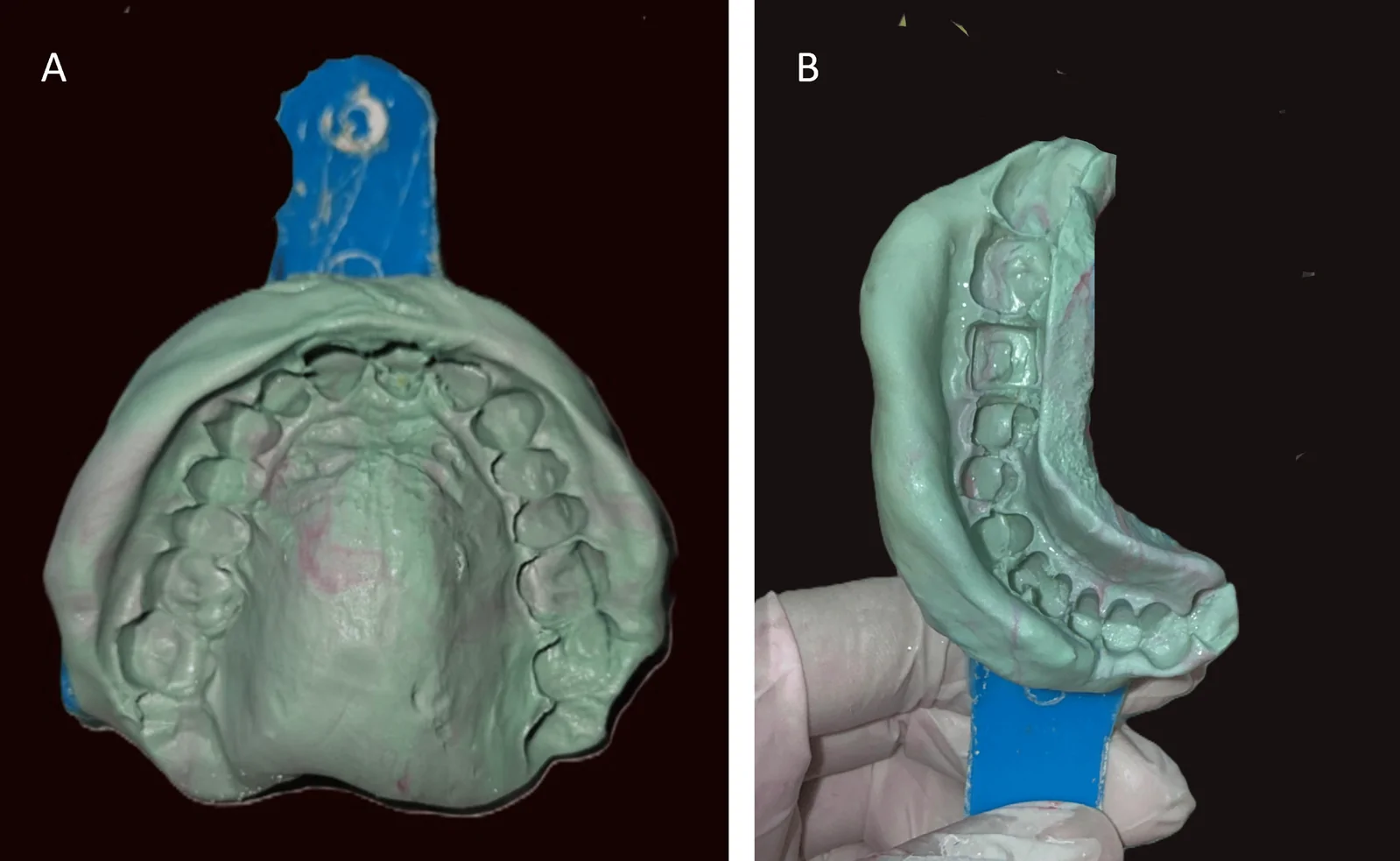
Maxillary and mandibular preliminary impressions. (A) Maxillary impression. (B) Mandibular impression

A maxillary palatal ramp prosthesis was fabricated using heat-polymerized acrylic resin (Dental Products of India (DPI) Heat Cure Acrylic Resin; Dental Products of India Ltd., Mumbai, India). The palatal ramp was designed to provide a guiding surface for the residual mandibular segment and was fabricated in relation to the desired mandibular position. The mandibular segment was manually guided toward a more favourable intercuspal relationship, and the palatal ramp was adjusted to provide appropriate contact with the mandibular teeth during functional movements. The ramp was checked intraorally to evaluate the guiding occlusal contacts, adequate retention, patient comfort, and unobstructed mandibular opening and closing.

A touch sensor was incorporated within the palatal aspect of the ramp at the intended contact region with the mandibular teeth. The sensor was connected to a wireless transmitter-receiver system and an external buzzer powered by a coin-cell battery. The electronic components, including the transmitter-receiver and battery, were incorporated within the prosthetic structure and enclosed to prevent direct contact with the oral tissues. Contact between the mandibular teeth and the designated area of the palatal ramp activated the buzzer, thereby providing immediate auditory feedback when the patient reached the intended mandibular position. The incorporation and assembly of the electronic components were performed with assistance from an electrical engineering professional. During follow-up, the prosthesis and electronic assembly were clinically inspected for component displacement, leakage, mucosal irritation, or other complications; none were observed. Formal laboratory testing for battery-compartment leakage or mechanical retention was not performed. The components and completed sensor-enabled maxillary palatal ramp prosthesis are shown in Figure 4.

**Figure 4:**
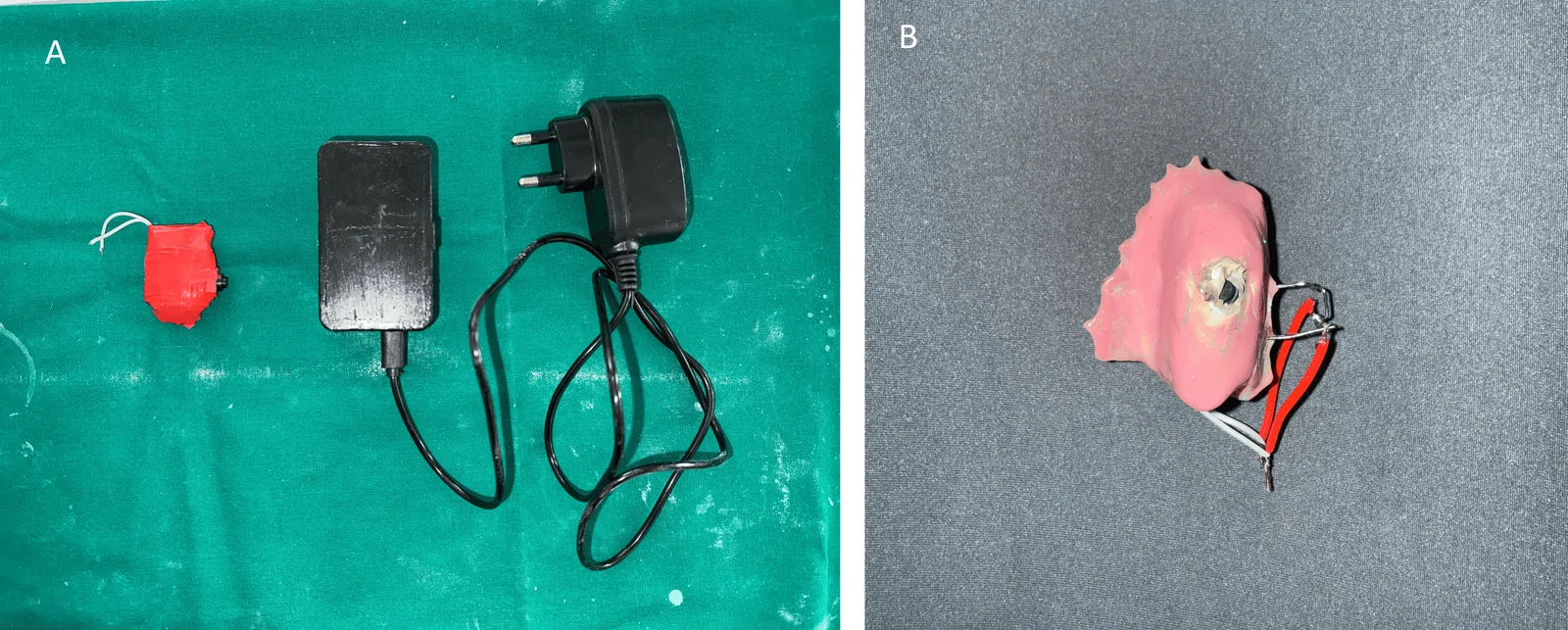
Components and assembly of the sensor-enabled maxillary palatal ramp prosthesis. (A) Touch sensor, wireless transmitter-receiver components, and external buzzer. (B) Completed sensor-enabled palatal ramp prosthesis with the components incorporated.

The completed prosthesis was inserted intraorally, and the ramp was clinically adjusted to obtain reliable sensor activation at the desired mandibular position. The prosthesis could be removed and cleaned using the routine cleaning procedures recommended for conventional removable prostheses, without requiring a special cleaning protocol. The patient was guided to move the mandible toward the intended position during functional movements. Activation of the buzzer provided immediate auditory feedback when the mandibular teeth contacted the designated sensor area, indicating that the desired mandibular position had been reached. This immediate feedback allowed the patient to recognize and repeatedly practice the desired mandibular position during mandibular guidance exercises associated with speaking, swallowing, and mastication. The feedback served as a positional training cue and was not intended as a quantitative measurement of mandibular movement or force. A clinical demonstration of the sensor-enabled palatal ramp prosthesis during mandibular positioning is provided in Video 1.

**Video 1:** Clinical demonstration of sensor-enabled palatal ramp prosthesis.

The prosthesis was delivered approximately one month after the initial consultation. The patient was instructed to perform regular mandibular guidance exercises during speaking, swallowing, and mastication while using the auditory feedback system. The patient was advised to use the auditory feedback system primarily during the prescribed rehabilitation exercises. During routine social activities and public settings, the patient did not routinely use the auditory feedback function, thereby avoiding repeated audible signals in public. The patient reported good acceptance of the auditory feedback system during the rehabilitation exercises and did not report discomfort or embarrassment related to the auditory signal. The sensor-enabled palatal ramp prosthesis was used as a temporary rehabilitation and training appliance for six months.

The coin-cell battery remained functional throughout the six-month rehabilitation period, and no battery replacement was required during this period. The patient was not required to perform battery replacement, and no battery-related service was necessary during the training period. The electronic assembly was checked during the scheduled clinical visits for proper functioning and any signs of malfunction. No interruption of auditory feedback due to battery depletion or electronic malfunction was observed during the six-month period. No predefined battery replacement interval was established because the battery remained functional throughout the six-month active training period; the electronic assembly was checked during scheduled clinical visits.

Follow-up evaluations were performed at 1, 3, 6, 12, and 18 months. The clinical outcome was evaluated using both quantitative and qualitative clinical assessments. Quantitative assessment included measurement of maximum mouth opening and mandibular deviation from the facial midline. Qualitative assessment included clinical evaluation of mandibular guidance, intercuspal relationship, mastication, and speech, together with the patient's subjective report of functional improvement. Compliance with the rehabilitation exercises was assessed through patient self-report and clinical observation during follow-up visits. Following completion of the six-month rehabilitation period, the patient was provided with the definitive prosthesis, and subsequent follow-up visits were performed to assess maintenance of mandibular guidance and functional performance. At the final 18-month follow-up, mandibular deviation had decreased from approximately 15 mm at baseline to 5 mm, while maximum mouth opening had increased from approximately 25 mm to 32 mm. The patient also demonstrated improved mandibular guidance and was able to achieve a more consistent intercuspal relationship. The oral mucosa was also clinically examined during follow-up for any suspicious changes or evidence of recurrence, considering the patient's previous squamous cell carcinoma and history of tobacco chewing; no clinically suspicious mucosal changes or evidence of recurrence were observed during the follow-up period. The clinical outcome following rehabilitation with the sensor-enabled palatal ramp prosthesis is shown in Figure 5.

**Figure 5:**
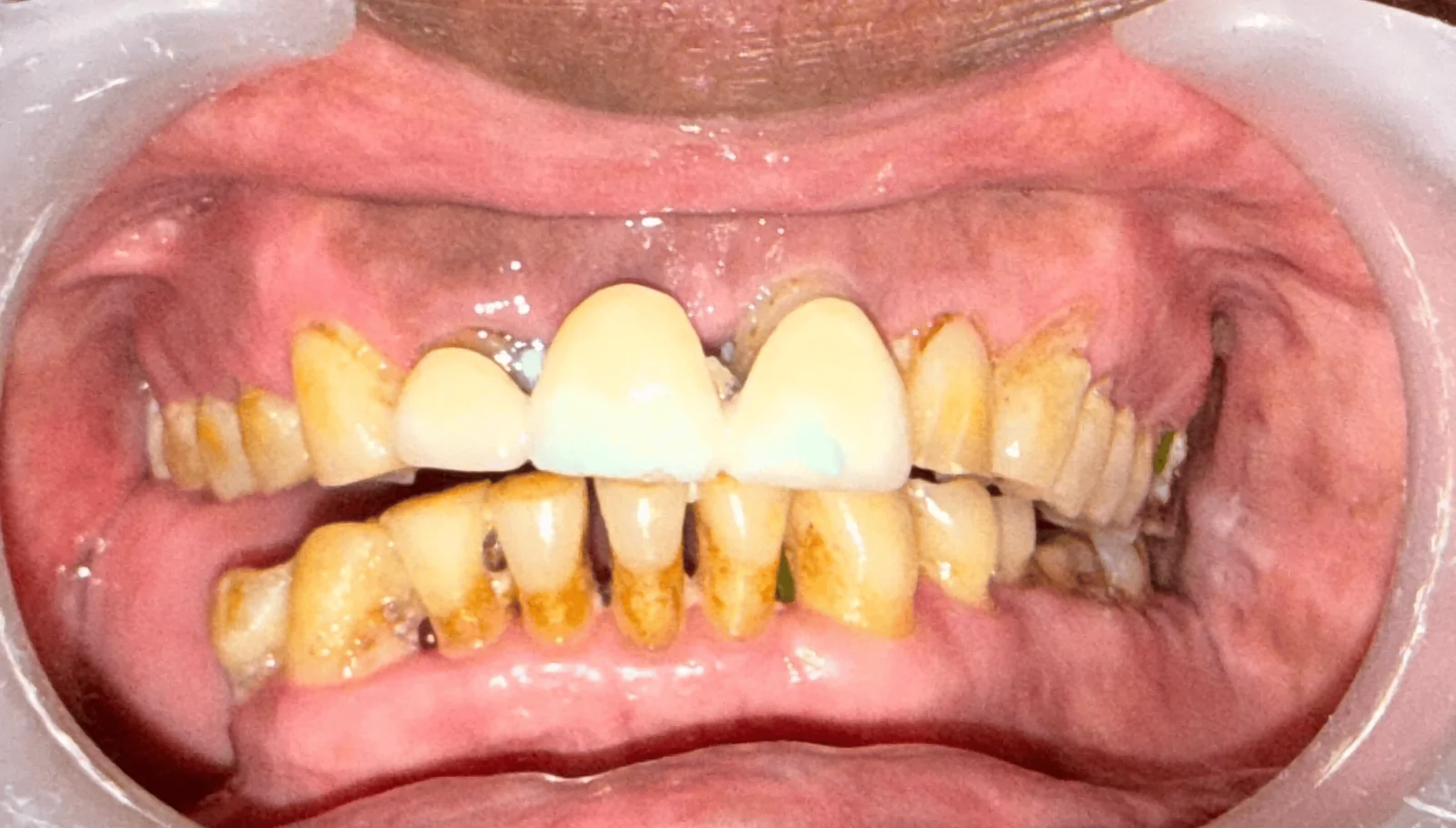
Clinical outcome at follow-up after rehabilitation with the sensor-enabled palatal ramp prosthesis.

Improvements in mastication and speech were observed clinically, and the patient reported good satisfaction with the prosthesis. No clinically suspicious mucosal changes or evidence of recurrence was observed during the follow-up period. No complications related to the prosthesis or sensor assembly were encountered during the follow-up period. Written informed consent was obtained from the patient for publication of this case report and the associated clinical images and video.

## Discussion

Functional rehabilitation following hemimandibulectomy remains a significant challenge because of mandibular discontinuity, muscular imbalance, and loss of proprioceptive guidance. These factors often result in mandibular deviation toward the resected side, making mastication, speech, swallowing, and maintenance of a stable occlusal relationship difficult. Early prosthodontic rehabilitation aims to reduce these functional impairments by retraining mandibular movements and improving neuromuscular coordination [4-8,10-16].

Guide flange prostheses, palatal ramp prostheses, and resection prostheses incorporating occlusal ramps are established treatment modalities for managing mandibular deviation following mandibular resection. A guide flange prosthesis provides mechanical guidance of the residual mandibular segment toward a more favourable position, whereas a palatal ramp prosthesis provides a guiding surface that assists the mandible in achieving a more favourable occlusal relationship during functional movements. Resection prostheses with occlusal ramps can similarly assist mandibular positioning by establishing a guiding occlusal relationship. Conventional mandibular guidance prostheses primarily depend on mechanical guidance, patient compliance, repeated functional exercises, and progressive neuromuscular adaptation [1,2,5-8,12,16,17]. Surgical reconstruction using vascularized bone grafts, with or without subsequent implant-supported rehabilitation, may restore mandibular continuity and provide a more definitive anatomical reconstruction; however, it involves greater surgical complexity, morbidity, and cost [3,4,7].

In the present case, surgical reconstruction was offered but declined by the patient due to financial constraints. Prosthetic rehabilitation was therefore selected according to the patient's clinical and socioeconomic circumstances. The sensor-enabled palatal ramp was designed as a temporary six-month rehabilitation and training appliance rather than a definitive prosthesis. It retained the mechanical guiding function of a conventional palatal ramp while incorporating an auditory feedback mechanism to assist the patient in recognizing the predetermined mandibular position during functional exercises.

The present case describes a modification of a conventional palatal ramp prosthesis by incorporating a touch sensor-based auditory biofeedback system. Correct contact between the mandibular teeth and the predetermined area of the palatal ramp activated the external buzzer, providing immediate auditory feedback. This allowed the patient to recognize when the desired mandibular position was achieved and to maintain that position during functional exercises such as speaking, swallowing, and mastication. Thus, the system provided real-time auditory positional feedback in addition to the mechanical guidance provided by the conventional ramp.

The touch sensor was incorporated within the palatal aspect of the ramp and connected to a wireless transmitter-receiver assembly powered by a coin-cell battery. The electronic components were incorporated within the prosthetic structure with attention to maintaining adequate retention, patient comfort, and functional use. During fabrication and clinical use, calibration of sensor sensitivity and positioning of the touch sensor were important to obtain reliable activation at the desired mandibular position. The incorporation of the electronic components also required management of the available space within the prosthesis to minimize additional bulk. No loose wiring, buzzer failure, or other technical failure was encountered during the six-month period of active use of the sensor-enabled appliance.

The patient was instructed to perform mandibular guidance exercises during speaking, swallowing, and mastication while using the auditory feedback system. The sensor-enabled appliance was used for approximately six months as a temporary rehabilitation and training device. During this period, the patient demonstrated improvement in mandibular guidance and functional performance. Following completion of the active training period, the patient was provided with definitive prosthetic rehabilitation. Subsequent follow-up visits were performed to assess maintenance of the corrected mandibular position and functional performance rather than continued use of the sensor-enabled training appliance.

At baseline, mandibular deviation was approximately 15 mm from the facial midline and maximum mouth opening was approximately 25 mm. At the final 18-month follow-up assessment, mandibular deviation was 5 mm and maximum mouth opening was 32 mm. Improvements in intercuspation, mastication, and speech were also observed clinically. These findings suggest an association between the rehabilitation protocol and improvement in mandibular guidance and functional measures; however, causality cannot be established from a single uncontrolled case.

Patient acceptance is an important consideration when an external auditory feedback system is incorporated into an intraoral prosthesis. In the present case, the patient reported good acceptance of the auditory feedback and did not report embarrassment, discomfort, or reluctance to use the appliance because of the buzzer. Compliance was assessed through patient self-report and clinical observation during follow-up. No prosthesis-related or sensor-related complications were observed during the reported period.

Compared with a conventional palatal ramp, the sensor-enabled design introduces additional components and therefore may involve greater cost, fabrication complexity, maintenance requirements, battery dependence, and potential risk of electronic malfunction. Sensor sensitivity may also require calibration, and the additional components may affect the available space, bulk, and retention of the prosthesis. Furthermore, prolonged dependence on auditory cues could potentially limit the transition from externally guided positioning to internally controlled mandibular movement. Therefore, the auditory feedback should be regarded as an adjunct to mechanical guidance and neuromuscular training rather than a substitute for the patient's own functional adaptation.

The safety and maintenance of sensor-integrated intraoral prostheses also require consideration. In the present case, the coin-cell battery remained functional throughout the approximately six-month active training period, and no battery replacement was required during this period. No battery-related leakage, dislodgement, mucosal injury, or interruption of the auditory feedback system was observed. Nevertheless, the use of electronic components within an intraoral prosthesis introduces potential risks related to battery depletion, component failure, retention, and accidental exposure of the battery assembly. These factors should be carefully considered during fabrication and follow-up, and appropriate enclosure and securing of the electronic components are essential. The prosthesis could be maintained using routine removable-prosthesis cleaning procedures, and no special cleaning-related complications were observed during the active six-month training period.

The present approach should be considered within the broader spectrum of rehabilitation options for hemimandibulectomy defects. Guide flange and conventional palatal ramp prostheses provide established mechanical methods of mandibular guidance, while surgical reconstruction may restore anatomical continuity and facilitate more definitive rehabilitation in appropriately selected patients [3-8,10-17]. The sensor-enabled palatal ramp does not replace these established approaches; rather, it represents a sensor-assisted modification intended to provide additional auditory feedback during the rehabilitation and training phase. Its use may be particularly relevant when prosthetic guidance is selected, and additional feedback is considered beneficial for patient training.

The concept of incorporating sensor-assisted feedback into prosthodontic rehabilitation has potential for further development. However, claims regarding the novelty or superiority of this approach should be interpreted cautiously because the present report describes a single clinical application. Future developments may include pressure-sensitive sensors, force-mapping systems, mandibular movement tracking, smartphone-based rehabilitation applications, and other digital feedback systems capable of assisting individualized rehabilitation. Such technologies may provide opportunities for more objective monitoring of mandibular function during rehabilitation.

The primary limitation of this report is that it describes a single patient without a control group or comparison with conventional palatal ramp therapy. Although mandibular deviation and maximum mouth opening were measured clinically, the functional outcomes, such as mastication and speech, were assessed primarily through clinical observation, and compliance was assessed through patient self-report and clinical observation. The results therefore cannot be generalized to all patients with hemimandibulectomy defects. In addition, the sensor-enabled appliance was used only during the six-month training period, and the subsequent follow-up was intended to assess maintenance of mandibular position and functional performance after completion of active sensor-assisted rehabilitation. Further prospective studies involving larger patient populations, standardized objective outcome measures, and comparison with conventional mandibular guidance prostheses are required to determine the effectiveness, reliability, and broader applicability of this approach.

## Conclusions

The sensor-enabled palatal ramp prosthesis described in this report represents a sensor-assisted modification of conventional mandibular guidance therapy for rehabilitation following hemimandibulectomy. The incorporation of a touch sensor-based auditory biofeedback system provided immediate auditory reinforcement when the predetermined mandibular position was achieved during functional training. In the present case, the six-month use of the sensor-enabled training appliance was associated with improvement in mandibular guidance, with mandibular deviation decreasing from 15 mm at baseline to 5 mm at the final 18-month assessment and maximum mouth opening increasing from 25 mm to 32 mm. Improvements in intercuspation, mastication, and speech were also observed clinically, with good patient acceptance and no prosthesis- or sensor-related complications reported during the follow-up period. These findings suggest that sensor-assisted auditory feedback may have potential as an adjunct to conventional mechanical mandibular guidance during the rehabilitation and training phase; however, a causal relationship cannot be established from this single uncontrolled case. Further prospective clinical studies involving larger patient populations, standardized objective functional outcome measures, and comparison with conventional mandibular guidance prostheses are required to determine the effectiveness, reliability, and broader clinical applicability of this approach.
